# Performance Comparison of Bench-Top Next Generation Sequencers Using Microdroplet PCR-Based Enrichment for Targeted Sequencing in Patients with Autism Spectrum Disorder

**DOI:** 10.1371/journal.pone.0074167

**Published:** 2013-09-16

**Authors:** Eriko Koshimizu, Satoko Miyatake, Nobuhiko Okamoto, Mitsuko Nakashima, Yoshinori Tsurusaki, Noriko Miyake, Hirotomo Saitsu, Naomichi Matsumoto

**Affiliations:** 1 Department of Human Genetics, Yokohama City University Graduate School of Medicine, Yokohama, Japan; 2 Department of Medical Genetics, Osaka Medical Center and Research Institute for Maternal and Child Health, Osaka, Japan; Rikagaku Kenkyūsho Brain Science Institute, Japan

## Abstract

Next-generation sequencing (NGS) combined with enrichment of target genes enables highly efficient and low-cost sequencing of multiple genes for genetic diseases. The aim of this study was to validate the accuracy and sensitivity of our method for comprehensive mutation detection in autism spectrum disorder (ASD). We assessed the performance of the bench-top Ion Torrent PGM and Illumina MiSeq platforms as optimized solutions for mutation detection, using microdroplet PCR-based enrichment of 62 ASD associated genes. Ten patients with known mutations were sequenced using NGS to validate the sensitivity of our method. The overall read quality was better with MiSeq, largely because of the increased indel-related error associated with PGM. The sensitivity of SNV detection was similar between the two platforms, suggesting they are both suitable for SNV detection in the human genome. Next, we used these methods to analyze 28 patients with ASD, and identified 22 novel variants in genes associated with ASD, with one mutation detected by MiSeq only. Thus, our results support the combination of target gene enrichment and NGS as a valuable molecular method for investigating rare variants in ASD.

## Introduction

Recent advances in next generation sequencing (NGS) technologies combined with efficient gene enrichment, allows the comprehensive resequencing of multiple known causative or associated genes in highly heterogeneous diseases. In addition, these technologies make it possible to perform resequencing more inexpensively and rapidly than the conventional Sanger method. Higher sequencing accuracy due to the deeper achievable coverage with the aid of improved bioinformatic analysis is expected as well. Different bench-top next generation DNA sequencers are currently available for target resequencing. Each NGS machine adopts specific technologies, thus the property and/or quality of sequence reads is likely different. However there is little comparative evidence on the data quality between sequencers used in human gene analysis.

Autism spectrum disorder (ASD) is a complex disorder with several hundred associated loci, following a polygenic mode of inheritance [1]. It is relatively common, with a prevalence of 1.1% [2], and is typically a child-onset disorder characterized by impaired social interactions, communication deficits, and restricted and repetitive behaviors [3]. It is known to be highly heritable, yet the majority of its heritability is so far unresolved [4]. Previous studies suggest a genetic contribution, consisting of both common and rare alleles, accounts for a portion of ASD risk, with a heritability of 38–90% [4]–[8]. Considering the frequency and socio-economic impact of ASD, verification of the actual heritability of ASD is of importance. Common single-nucleotide variants (SNVs) have been reported as a major source of ASD risk, with the heritability exceeding 40% [7]. However, their impact on ASD development is relatively small in each case, with an estimated odds ratio (OR) <1.2 [9]. Conversely, rare variants occurring *de novo* or inherited are assumed to affect ASD risk as well [1], [10]–[13]. Recent work revealed a larger effect of *de novo* SNVs, although they accounted for only a small portion of overall ASD risk, with an estimated 10% contribution to ASD risk [10]–[13]. Recently, an additive 5% contribution to ASD risk was reported in rare complete knockouts, derived from inheriting rare recessive variations [14]. To further explore the missing ASD risk heritability, a promising approach would be to comprehensively identify rare variants that have additive gene effects or show a multigenic epistatic contribution.

Here we have developed a rapid, cost-effective and comprehensive analysis workflow for detecting rare variants in ASD patients. We screened 62 known ASD associated genes using microdroplet PCR-based technology, together with the Ion Torrent Personal Genome Machine (PGM) and MiSeq platforms. To validate the systems, we sequenced 10 positive controls with other diseases and 28 ASD patients. Sequencing data produced by the two sequencers were compared, demonstrating successful identification of positive control variants and novel SNVs associated with ASD.

## Materials and Methods

### Ethics statement

Written informed consents were obtained from all patients or their parents. Experimental protocols were approved by the Committee for Ethical Issue at Yokohama City University School of Medicine.

### Patients

A total of 28 ASD patients, diagnosed according to DSM IV-TR criteria, and 10 patients with other identified diseases with known mutations in one of the target genes, were used for this study. DNA was obtained from peripheral blood leukocytes.

### RainDance library preparation and DNA enrichment

The RainDance ASDSeq™ Research Screening Panel was provided by RainDance Technologies™ (Lexington, MA, USA). The RainDance ASDSeq™ panel is a genetic screening tool that offers >92% coverage of 62 genes containing known mutations associated with ASD. The library contains 2349 amplicons ranging in size from 167 to 600 bp and covering a 1034 kb region. Coverage includes all exons for each gene plus 50 bp up- and downstream of each exon, to capture intron/exon splice junctions, as well as 1 kb of both the 5′ promoter region and 3′ UTRs. The panel includes both autosomal and X-linked genes.

A total of 2.5 µg of genomic DNA was used for DNA enrichment. The primer library and a template mix, including 1.5 µg of fragmented genomic DNA and all the PCR reaction components except the primers, were loaded on the RainDance for PCR droplet preparation, according to the manufacturer’s instructions. Samples were run on the RDT 1000 machine and PCR droplets were generated. The PCR droplets were amplified under the following conditions: 94°C for 2 min, then 54 cycles of 94°C for 30 sec, 54°C for 30 sec and 68°C for 60 sec, followed by 68°C for 10 min and 4°C for holding. After amplification, the PCR droplets were broken to release the amplicons. The amplicons were purified and quantified using the 2100 Bioanalyzer (Agilent Technologies, Santa Clara, CA, USA). The ends of the DNA fragments were repaired at 25°C for 30 min using New England BioLabs End Repair Module (New England BioLabs, Ipswich, MA, USA), followed by purification using Qiagen MinElute columns (Qiagen, Valencia, CA, USA). The PCR fragments were concatenated at 20°C for 30 min using NEB Quick Ligation Kit (New England BioLabs). The ligated products were purified using the Qiagen MinElute columns and fragmented using a Covaris S2 machine (duty cycle 10%, intensity 5, cycle/burst 200, total time per treatment 430 s).

### Sequencing using ion torrent PGM and data processing

Library preparation was carried out using the Ion Plus Fragment Library Kit, with 50 ng of amplicons. Adapter ligation, nick repair and amplification were performed as described in the Ion Torrent protocol (Ion Plus Fragment Library Kit; Part Number 4471989 Rev. B; Life Technologies, Grand Island, NY, USA). The Agilent 2100 Bioanalyzer (Agilent Technologies) and associated High Sensitivity DNA kit (Agilent Technologies) were used to determine quality and concentration of the libraries. Emulsion PCR and enrichment steps were carried out using the Ion OneTouch™ Template Kit (Life Technologies) and associated protocol (Part Number 4472430 Rev. C). Sequencing of the amplicon libraries was carried out on the Ion Torrent PGM system using 316 or 318 chips, and barcoding with Ion Xpress™ Barcode Adapters 1–16 Kit (Life Technologies). The Ion Sequencing Kit v2 (Life Technologies) was used for all sequencing reactions (expected read length was 100 bp), following the recommended protocol (Part Number 4469714 Rev. B). After sequencing, reads were mapped to hg19 using Torrent Mapping Alignment Program (TMAP). TMAP is a customized mapping tools for sequencing data generated by PGM, ignoring the indel calls around homopolymer stretch to reduce the hundreds of false negative calls. Torrent Suite 2.0 and/or 3.2 were used for all analyses. Coverage depth was calculated using Torrent Coverage Analysis. SNVs and small insertions/deletions (indels) were identified using the Torrent Variant Caller. Common variants (MAF ≥1%) registered in dbSNP135 (http://www.ncbi.nlm.nih.gov/projects/SNP/) without a flag as clinically associated, or ones in the lower versions of dbSNP, were filtered out. Filter-passed variants were annotated using ANNOVAR [15] and a custom pipeline. In order to compare the ability of mutation detection, reads of positive controls were aligned to GRCh37 with Novoalign v3.00 (Novocraft Technologies, Selangor, Malaysia) with the parameters for PGM and Local realignments around indels and base quality score recalibration were performed using the Genome Analysis Toolkit (GATK) v1.5–21 [16]. SNVs and small indels were identified using the GATK UnifiedGenotyper.

### Sequencing using MiSeq and data processing

The same amplicons were sequenced on the Illumina MiSeq sequencer, using the SureSelect^XT^ Reagents (Agilent Technologies) protocol, with 50 ng input material. Each multiplex library pool was sequenced on an Illumina MiSeq for 150 cycles from each end, plus a 6 base-index sequence read, using the MiSeq Reagent Kit (Illumina, San Diego, CA, USA). Image analysis and base calling were performed using sequence control software with real-time analysis, and Consensus Assessment of Sequence and Variation (CASAVA) software v1.8 (Illumina). Reads were aligned to GRCh37 with Novoalign v2.08 (Novocraft Technologies), and Local realignments around indels and base quality score recalibration were performed using the GATK v1.5–21 [16]. SNVs and small indels were identified using the GATK UnifiedGenotyper, and filtered according to the Broad Institute’s best-practice guidelines v3. Common variants (MAF ≥1%) registered in dbSNP135 (http://www.ncbi.nlm.nih.gov/projects/SNP/) without a flag as clinically associated, or ones in the lower versions of dbSNP, were filtered out. Filter passed variants were annotated using ANNOVAR [15] and a custom pipeline.

### Quality validation of sequence reads

For quality comparison, we combined sequencing data from four random samples obtained by either PGM or MiSeq and evaluated the average quality of data from multiple samples. Box plots for base-call quality of combined runs from each sequencer were generated using fastqc software (Babraham Bioinformatics, Cambridge, UK). To count the number of single nucleotide polymorphisms (SNPs) and short indels in our combined sequencing data, we used samtools mpileup command with the minimum mapping quality assignment option. We excluded calls with either a depth ≤10 or genotype quality ≤30.

### Validation of novel variants

PolyPhen-2 (http://genetics.bwh.harvard.edu/pph2/), SIFT (http://sift.jcvi.org/www/SIFT_BLink_submit.html), MutationTaster (http://www.mutationtaster.org/) and Genomic Evolutionary Rate Profiling (GERP) [17] were used to evaluate SNVs in terms of sequence conservation, chemical change and likelihood of pathogenicity. The Human Gene Mutation Database (Biobases, Wolfenbuettel, Germany; (https://portal.biobase-international.com/hgmd/pro/start.php) was used for determining if variants were previously reported.

### Sanger confirmation of variants detected by next-generation sequencing

Possible pathological variants were confirmed by Sanger sequencing using an ABI 3500×l or ABI 3100 autosequencer (Life Technologies), according to the manufacturer’s protocol. Sequencing data was analyzed using sequence analysis software version 5.1.1 (Applied Biosystems, Foster City, CA, USA) and Sequencher 4.10-build 5828 (GeneCodes Corporation, Ann Arbor, MI, USA).

### Statistical analysis

All statistical analyses were carried out using SPSS Statistics 19 (IBM, NY, USA). The carrier frequency of each novel SNV was compared between ASD patients and in-house 212 normal Japanese controls using Fisher’s exact test. *p*<0.05 was considered statistically significant.

## Results

### Sequencing yields and targeting efficiency

The targeted NGS panel was designed to amplify all exons of the 62 known ASD associated genes (Table S1). To validate the performance of RainDance sample enrichment and our chosen NGS systems, ten positive controls, each with a mutation in either *NSD1* (c.3958C>T, c.5177C>T, c.5179G>C, c.6499T>C), *MECP2* (c.243_244insC, c.316C>T), *CASK* (c.277_288del), *SCN1A* (c.342_344delinsAGGAGTT, c.4313T>A) or *CDKL5* (c.145G>A) were used. Our workflow strategy is summarized (Table 1). NGS after target enrichment yielded an average of 295.97 (PGM-TMAP), 201.73 (PGM-Novoalign) and 469.42 (MiSeq) Mb of sequence, in which 96.8% (PGM-TMAP), 78.8% (PGM-Novoalign) and 75% of reads were mapped to the genome, and 26.7% (PGM-TMAP), 28.3% (PGM-Novoalign) and 22.7% were mapped to the targeted regions, by PGM and MiSeq, respectively (Table 2). The percentage of mapped bases was greater in PGM-TMAP than in PGM-Novoalign, while the ones in PGM-Novoalign and MiSeq were similar. On-target rate was also similar and generally low in these data. The total coverage of all targeted bases was on average for PGM (TMAP), 93.7% at 10× and 85.9% at 20×, with a mean read depth of 63×, and for MiSeq, 96.8% at 10× and 93.2% at 20×, with a mean read depth of 95× (Table 2). The complete coverage information on the differences between PGM and MiSeq is presented in Table 2. The mean depth of coverage on genes across all samples ranged from 21× for *PTCHD1* to 237× for *NHS*, with an average of 95× by MiSeq. Despite the high mean read depth and target region coverage, several exons including exon 15 of *NIPBL*, exon 43 of *RELN*, exon 2 of *BRAF*, exon 7 of *PTEN*, exon 10 of *SLC6A4*, exon11 of *SHANK3*, exon 43 of *DMD*, exon 8 of *CASK*, exon 36 of *MED12* and exon 2 of *L1CAM*, had no mapped reads from either sequencer. These unmapped regions may be due to sequence complexity, problematic library synthesis necessitating the use of a concatenation step for sample preparation, or unusual GC content of the fragments for the enrichment system. Exon 11 of *SHANK3* has a very high GC content (80%), while exons 2, 43, 15, and 43, of *BRAF*, *DMD*, *NIPBL*, and *RELN*, respectively, have a very low GC content (<35%), and consequently no mapped reads in the NGS data.

**Table 1:** Strategy for validation of RainDance sample enrichment and NGS methods.

	PGM	MiSeq
Number of samples	10	10
Sample enrichment	RDT1000*	RDT1000*
Sequence generated	100 bp single-end** (316 chip/318 chip)	150 bp pair-end (Miseq Reagent Kit)
Mapping	TMAP v2.0.1/Novoalign	Novoalign
SNP/indel identification	Variant caller/GATK	GATK
Annotation	ANNOVAR	ANNOVAR

* The sequencing library used was the RainDance ASDSeq™Research Screening Panel.

** PGM provided the protocol for paired-end sequencing in the end of 2011, only for optional.

**Table 2:** Comparison between PGM and MiSeq sequencing performance in 10 positive controls.

	PGM		MiSeq
	TMAP	Novoalign	
Average total number of bases (Mb)	295.97	201.73	469.42
Average read length (base)	116	116	150
% mapped on human genome	96.8%	78.8%	75%
% on target regions	26.7%	28.3%	22.7%
Mean depth of coverage	63	57	95
% of target regions at>10-fold coverage	93.7%	92.1%	96.8%
% of target regions at>20-fold coverage	85.9%	82.0%	93.2%

### Comparison of sequencing quality

The mean base-call quality score obtained from MiSeq was high through entire reads, with a score >30 (Figure S1A, B). The dispersion of scores among reads at specific positions was relatively small. Conversely, the mean base-call quality score obtained from PGM was >25 at the beginning of reads, but gradually decreased to around 20, at approximately base position 100. The dispersion of scores among reads was larger than those obtained using MiSeq. n addition, read lengths produced by each sequencer were different. With MiSeq, all reads had the expected length of 151 bases, whereas with PGM, read lengths were widely distributed from 60 to 150 bp long, although the expected read length was 100 bp (Figure S1C).

Overall, it appeared that the MiSeq output sequences had a higher base-call quality, but it was difficult to compare the scores derived from each sequencer, as PGM and MiSeq adopt different scoring systems for evaluating base-call quality. MiSeq uses Phred [18], while PGM uses a unique Phred-like system consisting of six predictors whose quality values are correlated with the probability of a base miscall. Therefore we compared the mapping quality of each read from both sequencers, as both sequencers adopt the same scoring system for mapping quality [19]. We summed up the total number of reads with a mapping quality >40 and reads <40, and found 94.5% (MiSeq) and 71.2% (PGM) of aligned reads had a mapping score >40 (Figure S1D).

Next we compared the number of indel calls detected by PGM and MiSeq, in the combined data from four individuals randomly chosen (Table S2). With PGM, 9685 SNPs or indels were called, with 5544 indels calls (57.2%). The frequency of indels was calculated as 1.34 per 1 kb per sample. With MiSeq, 3818 SNPs or indels were called, with 395 calls (10.3%) being indels. The frequency of indels was calculated as 0.096 per 1 kb per sample. After filtering the SNP and indel call with a mapping quality >40, and comparing again, 5288 indels out of 7574 total calls (69.8%) were detected with PGM, while 386 indels out of 3553 total calls (10.9%) were detected with MiSeq, leading to an expected frequency of 1.27 indels per 1 kb per sample (PGM) versus 0.093 indels per 1 kb per sample (MiSeq).

### Confirmation of variant detection

The ability of PGM and MiSeq to efficiently detect various mutations, including point mutations and small indels, was tested using previously Sanger-confirmed mutations in variant-positive samples (Table 3). The variant-positive samples included all types of variants, including missense, small insertion, small deletion and small indel variants, in the genes *SCN1A*, *NSD1*, *MECP2*, *CDKL5* and *CASK* (Table 3). Some of the insertion and indel variants detected by NGS are shown (Figure S2A, B). All confirmed variants had a coverage of at least 8× reads, and a mutant allele percentage of 33–62% for heterozygous or 83–100% for hemizygous variants (Table 3). The mutation detection rate was either 70% (PGM using standard analysis software of TMAP and Variant Caller) or 100% (MiSeq). With PGM, the variant located near the homopolymer could not be detected because of PGM’s high frequency of homopolymer sequencing errors [20], [21]. When using TSv3.2 for PGM data analysis, one out of four mutations not identified by TSv2.0, were additionally detected. In order to analyze on the same analytical platforms, sequence data of PGM were also processed using Novoalign for mapping and GATK for variant calling. The mutation detection rate differed significantly between platforms (TMAP-Variant Caller and Novoalign-GATK) (Table 3). Respective PGM data, displayed in the Integrative Genomics Viewer (IGV) [22], showed an increase in sequence mismatch patterns at amplicon ends.

**Table 3:** Validation of our chosen NGS methods for mutation detection.

					Detected by			Coverage			Mutant allele (%)		
Sample	Sex	Chr	Gene	Mutation	PGM1)	PGM2)	MiSeq	PGM1)	PGM2)	MiSeq	PGM1)	PGM2)	MiSeq
1	F	2	*SCN1A*	c.342_344delinsAGGAGTT	−	−	+	13	n.a.	91	n.a.	n.a.	44
2	F	2	*SCN1A*	c.4313T>A (p.M1438K)	+	+	+	31	42	48	33	31	38
3	M	5	*NSD1*	c.3958C>T (p.R1320X)	+	−	+	34	n.a.	50	62	n.a.	40
4	M	5	*NSD1*	c.5177C>T (p.P1726L)	+	−	+	37	n.a.	93	38	n.a.	46
5	M	5	*NSD1*	c.5179G>C (p.A1725P)	+	−	+	55	n.a.	62	47	n.a.	50
6	M	5	*NSD1*	c.6499T>C (p.C2167R)	+	−	+	77	n.a.	223	46	n.a.	54
7	F	X	*MECP2*	c.243_244insC	−	−	+	18	n.a.	123	n.a.	n.a.	41
8	F	X	*MECP2*	c.316C>T (p.R106W)	+	−	+	60	n.a.	76	42	n.a.	47
9	M	X	*CDKL5*	c.145G>A (p.E49K)	−	−	+	8	n.a.	46	n.a.	n.a.	100
10	M	X	*CASK*	c.227_228del	(+)	+	+	35	47	112	83	81	97

F, Female; M, Male; Chr, Chromosome; +, Detected; −, Not detected; (+), Mutation only detected by TSv3.2, and not by TSv2.0.; n.a., Not applicable;

1) Reads were mapped by TMAP and SNVs and indels were identified using the Torrent Variant Caller.

2) Reads were mapped by Novoalign v3.00 and SNVs and indels were identified using the GATK v1.5–21.

### Validation of the RainDance ASD panel for detecting novel mutations in ASD patients

RainDance targeted resequencing was obtained on a total of 28 ASD patients, with a mean total sequence length of 273 or 446 Mb, and an average read depth of approximately 65× or 115×, for PGM and MiSeq, respectively (Table 4). After filtering by dbSNP135, a total of 98 (PGM) and 62 (MiSeq) variants were discovered following RainDance target enrichment. Of these, 62 (PGM) and 46 (MiSeq) were nonsynonymous SNVs (Table S3). Under a rare variant hypothesis, variants were filtered to exclude common variants (MAF ≥1%), using the Exome Variant Server from the NHLBI Exome Sequencing Project and an internal dataset of 212 control exomes from the Japanese population. Although c.878C>T (p.S293F) in *SLC6A4* was detected in 4/212 control exomes (MAF = 0.01%), we chose not to remove this SNV, since it has been functionally proven to disrupt serotonin transporter activity [23]. We validated a total of 57 (PGM) and 30 (MiSeq) SNVs. These SNVs were confirmed by Sanger sequencing, with 21 (PGM) and 22 (MiSeq) shown to be true positives (Table S3). In contrast, after filtering to exclude common variants, no indel mutations were detected by either PGM or MiSeq. All 21 SNVs detected by PGM were also detected by MiSeq. We analyzed the ability of each platform to detect variants and found that both platform was able to identify true variants, but PGM produced many false variant calls. The true positive call rates in the entire coding region were 36.8% (PGM) and 73.3% (MiSeq) (Table S3). We inspected each false positive calls in PGM and MiSeq using IGV to evaluate what kind of errors they were. In PGM, 27/36 calls (75%) had low depth, 21/36 calls (58.3%) had calls at respective read end, 14/36 calls (38.8%) were located near homopolymers, and 1/36 calls (2.7%) had PGM specific low quality error. In MiSeq, 5/8 calls (62.5%) had calls at respective read end and 3/8 calls (37.5%) had MiSeq specific errors. (Table S3).

**Table 4:** Comparison between PGM and MiSeq sequencing performance in 28 ASD patients.

	PGM	MiSeq
Average of total number of bases (Mb)	273.06	445.99
% on target regions	30.20%	25.60%
Mean depth of coverage	65	115
% of target regions at>10-fold coverage	92.70%	95.50%

### Candidate rare SNVs associated with ASD

We identified 22 rare SNVs in 28 patients with ASD (Table 5). Clinical features of the patients with these rare SNVs were demonstrated (Table S4). We considered some to be disease causing, as they are the same mutations previously reported in patients with different diseases that accompany autistic features, namely, c.4612G>A (p.V1538I) in *SCN1A*, identified in a patient with Dravet syndrome [24], and c.878C>T (p.S293F) in *SLC6A4*, identified in a patient with serotonin transporter deficiency [23]. The c.7880G>A (p.R2627Q) mutation identified in *CHD7* was not the same mutation, but was found at the same position, as the one detected in a patient with CHARGE syndrome [25]. Of these three patients, parent samples were only available for the patient with the *SLC6A4* mutation, and the mutation was shown to be inherited from a mother with no autistic features.

**Table 5:** Rare SNVs identified with amino acid changes and computational predictions of pathogenicity.

Gene	Accession No.	Nucleotide	MutationTaster	Polyphen21)	SIFT2)	GERP3)	HGMD4)	genotype (allele)				Patient
		: amino acid change		(Hum Div)				cases	controls	OR (95% CI)	*p* value	
*BRAF*	NM_004333	c.976A>G:p.I326V	polymorphism	0	0.71	−5.32	none	1/28 (1/56)	1/212 (1/424)	7.82(0.46–128.60)	0.22	A682
*CACNA1C*	NM_001129837	c.4706C>T:p.P1569L	polymorphism	0.001	**0.04**	2.39	none	1/28 (1/56)	0/212 (0/424)	n.d.	0.12	A681
*CHD7*	NM_017780	c.7880G>A:p.R2627Q	polymorphism	**0.997**	**0.01**	**5.56**	CHARGEsyndrome(R2627X )	1/28 (1/56)	0/212 (0/424)	n.d.	0.12	A634
*CHD7*	NM_017780	c.7652C>A:p.T2551N	polymorphism	0.01	0.31	**5.63**	none	1/28 (1/56)	0/212 (0/424)	n.d.	0.12	A447
*CNTNAP2*	NM_014141	c.1276C>A:p.L426I	polymorphism	**0.977**	**0.03**	**5.7**	none	1/28 (1/56)	0/212 (0/424)	n.d.	0.12	A479
*CNTNAP2*	NM_014141	c.1448G>A:p.R483Q	polymorphism	**0.991**	0.4	**5.07**	none	1/28 (1/56)	0/212 (0/424)	n.d.	0.12	A621
*DMD*	NM_004007	c.3479A>G:p.N1160S	polymorphism	**0.973**	0.22	1.36	none	1/28 (1/56)	0/212 (0/424)	n.d.	0.12	A668
*DMD*	NM_004007	c.2473A>G:p.M825V	polymorphism	0.026	0.36	3.87	none	1/28 (1/56)	0/212 (0/424)	n.d.	0.12	A668
*MID1*	NM_001193278	c.555G>A:p.M185I	polymorphism	**0.839**	**0.01**	**5.64**	none	1/28 (1/56)	0/212 (0/424)	n.d.	0.12	A669
*NIPBL*	NM_015384	c.1553C>T:p.T518I	polymorphism	0.275	**0**	**5.88**	none	1/28 (1/56)	0/212 (0/424)	n.d.	0.12	A681
*NRXN1*	NM_001135659	c.455G>A:p.G152D	**disease causing**	0	1	4.97	none	1/28 (1/56)	0/212 (0/424)	n.d.	0.12	A711
*NSD1*	NM_022455	c.2087T>C:p.V696A	polymorphism	0.189	**0.02**	3.94	none	1/28 (1/56)	0/212 (0/424)	n.d.	0.12	A464
*PNKP*	NM_007254	c.56C>T:p.A19V	polymorphism	0.026	0.27	4.55	none	3/28 (3/56)	1/212 (1/424)	**25.32** **(2.54–252.76)**	**0.005**	A627,A651,A674
*RAI1*	NM_030665	c.1148C>T:p.P383L	polymorphism	1	**0**	**5.55**	none	1/28 (1/56)	0/212 (0/424)	n.d.	0.12	A663
*RAI1*	NM_030665	c.4238T>C:p.M1413T	polymorphism	0.011	**0**	2.93	none	1/28 (1/56)	0/212 (0/424)	n.d.	0.12	A634
*RELN*	NM_005045	c.8915A>C:p.K2972T	**disease causing**	**0.996**	**0.03**	**5.89**	none	1/28 (1/56)	0/212 (0/424)	n.d.	0.12	A653
*SCN1A*	NM_001165963	c.4612G>A:p.V1538I	polymorphism	**0.89**	0.09	**5.76**	Dravetsyndrome	1/28 (1/56)	0/212 (0/424)	n.d.	0.12	A695
*SHANK3*	NM_033517	c.3169C>T:p.L1057F	polymorphism	0.232	0.27	3.19	none	1/28 (1/56)	0/212 (0/424)	n.d.	0.12	A668
*SLC6A4*	NM_001045	c.878C>T:p.S293F	**disease causing**	0	0.25	3	Serotonintransporterdeficency	1/28 (1/56)	4/212 (4/424)	1.93(0.21–17.87)	0.47	A674
*TSC2*	NM_000548	c.2032G>A:p.A678T	polymorphism	0.016	0.23	−0.706	none	1/28 (1/56)	1/212 (1/424)	7.82(0.48–128.60)	0.22	A647
*VPS13B*	NM_015243	c.820T>G:p.F274V	polymorphism	0.314	**0**	**5.45**	none	1/28 (1/56)	0/212 (0/424)	n.d.	0.12	A663
*VPS13B*	NM_017890	c.11960C>G:p.P3987R	polymorphism	0.437	0.06	3.85	none	1/28 (1/56)	0/212 (0/424)	n.d.	0.12	A619

1) PolyPhen2 scores close to 1 are likely to be pathogenic (highlighted in bold). HumDiv-trained Polyphen-2 assumes even mildly deleterious alleles as damaging to evaluate rare alleles potentially involved in complex phenotypes.

2) SIFT scores less than 0.05 are likely to be pathogenic (highlighted in bold).

3) GERP scores above 5 are highly conserved (highlighted in bold).

4) The Human Gene Mutation Database (HGMD) was searched to identify SNVs registered as disease causing mutations. Carrier frequencies of each SNV were statistically compared between ASD patients (cases) and in-house normal 212 controls (controls). Results are presented as odds ratio’s (OR) and p values. Pathogenic findings are shown in bold. CI, confidence interval; wt, wild type allele; mut, mutant allele; n.d., not determined.

Eighteen of the identified SNVs were not observed in 212 in-house Japanese control exomes, suggesting they may be strong candidates for ASD associated SNVs. The remaining four SNVs were also observed in control exomes; however, with a lower frequency than patients with ASD, leading to an OR of 1.93–25.32. In particular, c.56C>T (p.A19V) was detected significantly more frequently in patients with ASD than in controls (OR, 25.32; 95% confidence interval (CI), 2.54–252.76). The remaining SNVs did not reach statistical significance, likely due to the limited number of patients analyzed.

Based on web-based prediction software, 72.7% of the detected SNVs (16/22) were deemed pathogenic by either PolyPhen-2 (36.3%; 8/22 SNVs), SIFT (50%; 11/22 SNVs), or MutationTaster (13.5%; 3/22 SNVs). We annotated positions with their conservation as scored with the GERP. Mutations at highly conserved positions would be predicted to be functionally important (45.5%; 10/22 SNVs).

Five out of 28 patients had multiple SNVs (Table S5). Following the multigenic contribution theory in ASD [4], these could be associated with the onset or the severity of this disease.

## Discussion

We have developed an efficient workflow for detecting rare SNVs/indels in ASD associated genes using bench-top next generation sequencers with target gene enrichment. The evaluation and comparison of NGS devices are of recent interest to us. In this study we chose to compare the Ion Torrent PGM and Illumina MiSeq, which are currently the most popular NGS. The characteristics of the two devices are shown (Table S6). In this study, we compared the sequence yield and quality of these two NGS platforms, and showed a practical use for targeted resequencing of human genes.

Our comparison of two bench-top sequencers showed their yields were both greater than expected; however, the quality of sequence reads varied: better than expected through entire reads in MiSeq, while barely exceeding the minimum expected quality value with large discrete reads in PGM. Comparing the mapping quality of the two sequencers, which was calculated based on the same algorithm, the percentage of reads with a mapping quality ≤40 was markedly more in PGM than in MiSeq. Considering their target regions were the same, this difference reflects the difference of overall read quality from the two sequencers. Focusing on indel calls, we found an excess with PGM, compared to MiSeq. The number of MiSeq indel calls is reasonable, compared to the estimated error rate (0.11 to 0.08 per 1 kb) in conventional capillary sequencing of the human genome [26]. Even with filtering of the reads for low genotyping quality and depth, the excess indel calls in PGM did not decrease. As previously reported, excess indel calls or a lower read quality are considered to be largely due to homopolymers [20], [27]. This unique characteristics of PGM was reflected in the difference of mapped rates for PGM-generated data when using different mapping tools, TMAP or Novoalign. As shown in Table 2, the mapped rates of bases between PGM-generated data and MiSeq-generated data using Novoalign were similar, being reasonable since these two data were derived from the same sample libraries, while the one for PGM-generated data using TMAP was better. We assume this is because TMAP consider homopolymer-associated indel errors on mapping and could map more reads which standard mapping tools such as Novoalign could not. The difference in the mapped rates for PGM-generated data might affect the mutation detection rate. Based on the difference in mutation detection rates of positive controls in PGM-generated data with different pipelines (Table 3), custom mapping and the SNP/indel detection software, TMAP and Variant Caller, are necessary for the PGM workflow to reduce mapping errors without compromising detection sensitivity. Otherwise the number of false positive indel calls would be greatly increased.

Generally, target gene enrichment using the RDT machine worked well, but there were some disadvantages, including a relatively low on-target rate as shown in Table 2, and occasional sample enrichment failure. This may be partially due to the genomic complexity or a biased GC content of target regions. Alternatively, it may be due to the screening panel itself, which does not employ a tailed primer system using PCR amplification primers, therefore necessitating the use of the concatenation step for sample preparation.

In our workflow validation using ten positive controls, the mutation detection rate was lower with PGM than MiSeq. False negatives are largely due to the weakness in indel detection, implying not only excess false positive, but also increased false negative indel calls with PGM. Another typical false negative mutation identified with PGM was detected at amplicon ends. This may happen more readily with PGM as the read length is not as long as expected. On the other side, the higher coverage of the MiSeq data is expected due to the longer read lengths as well as paired end reads. With regards to SNV detection, both PGM and MiSeq showed high mutation detection rates (6/7 mutations, 85.7% in PGM vs. 7/7 mutations, 100% in MiSeq). Target resequencing of 28 patients with ASD identified 21 candidate SNVs in PGM versus 22 in MiSeq, again showing similar SNV variant detection abilities. Although there is a higher false-positive SNV call rate with PGM compared to MiSeq due to the same factors observed in positive control studies, At present it would be reasonable to apply PGM for SNV detection. Recent rapid up-dates of the device, chemistry and mapping/mutation detection software in PGM may potentially reduce these drawbacks in the near future.

ASD is a genetically heterogeneous disease, with a complex genetic architecture [4]. In particular, rare SNVs with a multigenic contribution are expected to play a specific role in the molecular pathogenesis of ASD. We have shown that our workflow works rapidly and inexpensively to address this issue by demonstrating our successful identification of novel candidate SNVs in ASD. Notably, A19V in *PNKP* was identified significantly more in patients with ASD than controls. PNKP (polynucleotide kinase 3′-phosphatase ) is a bi-functional enzyme that possesses both DNA 3′-phosphatase and DNA 5′-kinase activities, and associates with the single strand break repair machinery. Single strand break could be hazardous to the cell if left unrepaired, especially in central nervous system since frequently single strand breaks could happen [28]. *PNKP* is mutated in microcephaly, early-onset, intractable seizures and developmental delay (MCSZ), in autosomal recessive manner. Patients with MCSZ sometimes show variable behavioral problems, mainly hyperactivity [29]. Considering enzymatic activity of PNKP and its stability as reported [30], clinical symptoms of individuals with the heterozygous variant may not be as severe as MCSZ, however it could not be denied that possible decrease in enzyme activity or protein level of PNKP comparing to wild type might affect the normal development of central nervous system. It was implied that *PNKP* might be a candidate for ASD-related gene by copy number analysis previously [31]. We showed for the first time a candidate variant associated with ASD. Further study with larger samples is necessary to confirm its pathogenicity. It is also noted that there were some genes such as *CHD7, CNTNAP2, DMD*, and *RAI1*, in which two patients had private rare variants. It is speculated that the private variants of those might accumulate in ASD populations.

In conclusion, we present the comparison of two bench-top sequencers, PGM and MiSeq, through the newly developed workflow for the investigation of ASD. Analyzing larger sample sets may lead to unraveling of the missing heritability of ASD.

## Supporting Information

Figure S1 **Comparison of overall sequencing quality between PGM and MiSeq.** (A) Box plots of base-call quality scores across all bases obtained using PGM with a 316 chip (left panel) or MiSeq (right panel). Green and red areas indicate quality scores above 28 and below 20, respectively. Yellow boxes show upper and lower quartiles with whiskers indicating 10% and 90% quartiles. Red horizontal lines indicate the median value. Blue curves represent the mean quality scores. Quality scores are given based on the calculation of Phred-scaled quality values using q = -10log10(P), with P being the estimated error probability for that base-call. (B) Quality score distribution over all sequence reads obtained using PGM with a 316 chip (left panel in red) or MiSeq (right panel in blue). Combined data from four samples are displayed. Mean quality scores across all base-calls from a particular sequence, calculated as the Phred score, are shown on the X axis, and the number of reads with the specified mean sequence quality on the Y axis. (C) Distribution of read length from all sequence reads obtained using PGM with a 316 chip (left panel in red) or MiSeq (right panel in blue). Read lengths are shown on the X axis, and the number of reads with the specified read lengths on the Y axis. (D) Mapping quality from all sequence reads obtained using PGM with a 316 chip (red bars) or MiSeq (blue bars). The number of reads with a mapping quality of either <40 or ≥40 in each device (left panel). The percentage of reads with mapping quality ≥40 in each device (right panel). MQ, mapping quality.
(TIF)

Figure S2
**Comparison between PGM and MiSeq of mutations and sequence reads from positive control samples.** (A) The c.342_344delinsAGGAGTT mutation detected in Sample 1. (B) The c.243_244insC mutation detected in Sample 7. In both panels, data was obtained from either PGM (upper) or MiSeq (lower). Both the c.342_344delinsAGGAGTT mutation and the c.243_244insC mutation were not detected in PGM with neither PGM-TMAP-Variant Caller algorithm nor PGM-Novolign-GATK algorithm. Forward and reverse read strands are shown in pink and blue, respectively. Red and blue arrows indicate insertion and deletion positions, respectively, which were confirmed by Sanger sequencing. The horizontal bar indicates the deletion call, and symbols within the read strands (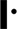 **·**) indicate insertion calls detected by either PGM or MiSeq. In (A) and (B), the true inserted sequence depicted by “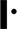 **·**” commonly detected by PGM and MiSeq is AACTCC and C, respectively. The DNA sequence surrounding a mutation is shown below the IGV graphics. WT, wild type; Pt, patient.(TIF)

Table S1 **RainDance ASDSeq™ Core Research Screening Panel.**
(PDF)

Table S2 **Summary of SNP/indel detection with PGM and MiSeq.**
(PDF)

Table S3 **Summary of target resequencing and prioritization.**
(PDF)

Table S4 **Clinical features of patients with novel SNVs.**
(PDF)

Table S5 **Multiple mutations detected in patients with ASD.**
(PDF)

Table S6 **Comparison of PGM and MiSeq analysis cost and expected yield.**
(PDF)
