# Supplementary figures and images for: Performance Comparison of Bench-Top Next Generation Sequencers Using Microdroplet PCR-Based Enrichment for Targeted Sequencing in Patients with Autism Spectrum Disorder
Source: PLoS One. 2013 Sep 16;8(9):e74167. doi: 10.1371/journal.pone.0074167 (PMC3774667; doi:10.1371/journal.pone.0074167)

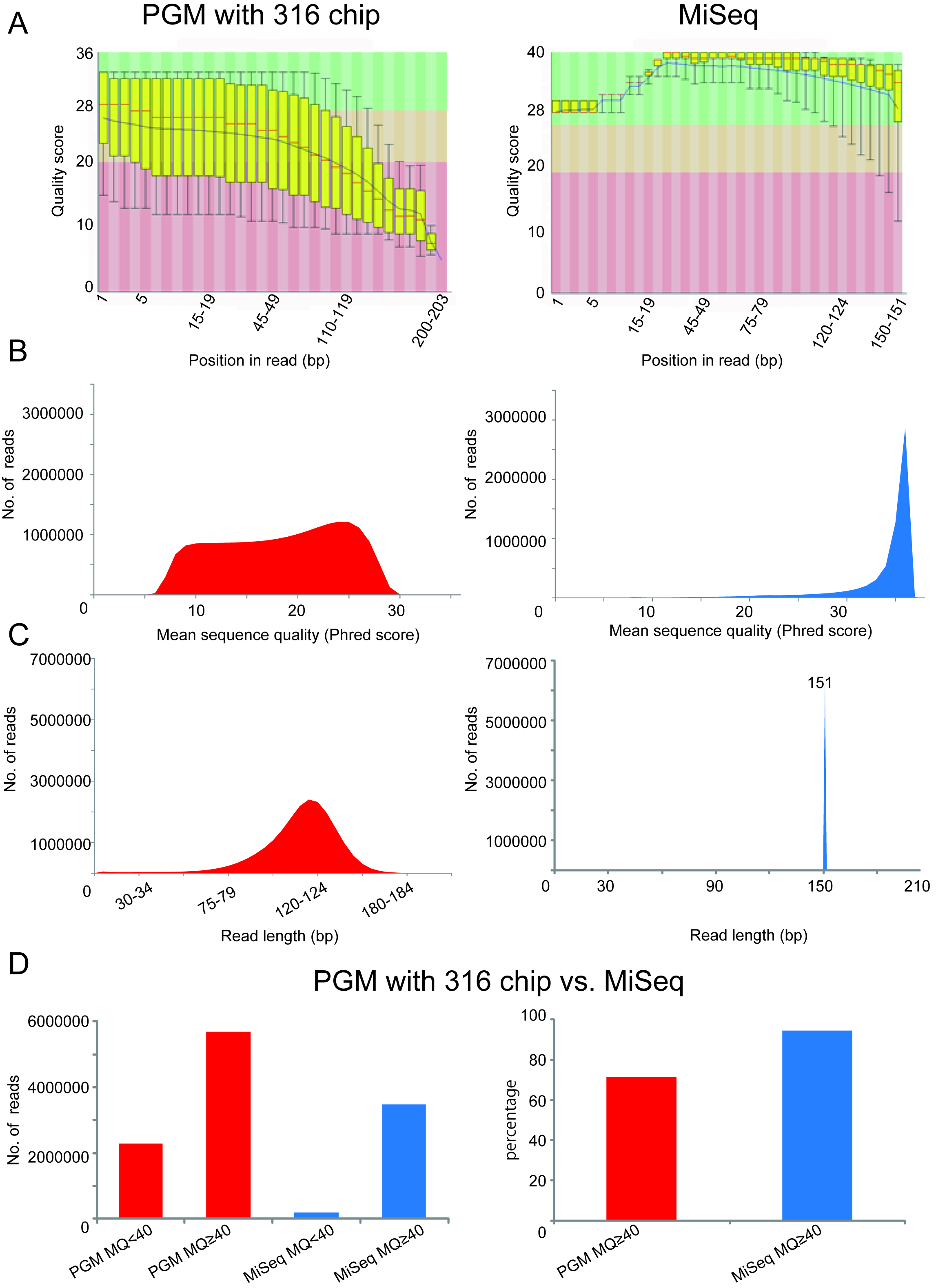

Supplement: Figure S1 — Comparison of overall sequencing quality between PGM and MiSeq. (A) Box plots of base-call quality scores across all bases obtained using PGM with a 316 chip (left panel) or MiSeq (right panel). Green and red areas indicate quality scores above 28 and below 20, respectively. Yellow boxes show upper and lower quartiles with whiskers indicating 10% and 90% quartiles. Red horizontal lines indicate the median value. Blue curves represent the mean quality scores. Quality scores are given based on the calculation of Phred-scaled quality values using q = -10log10(P), with P being the estimated error probability for that base-call. (B) Quality score distribution over all sequence reads obtained using PGM with a 316 chip (left panel in red) or MiSeq (right panel in blue). Combined data from four samples are displayed. Mean quality scores across all base-calls from a particular sequence, calculated as the Phred score, are shown on the X axis, and the number of reads with the specified mean sequence quality on the Y axis. (C) Distribution of read length from all sequence reads obtained using PGM with a 316 chip (left panel in red) or MiSeq (right panel in blue). Read lengths are shown on the X axis, and the number of reads with the specified read lengths on the Y axis. (D) Mapping quality from all sequence reads obtained using PGM with a 316 chip (red bars) or MiSeq (blue bars). The number of reads with a mapping quality of either <40 or ≥40 in each device (left panel). The percentage of reads with mapping quality ≥40 in each device (right panel). MQ, mapping quality. (TIF) [file pone.0074167.s001.tif]

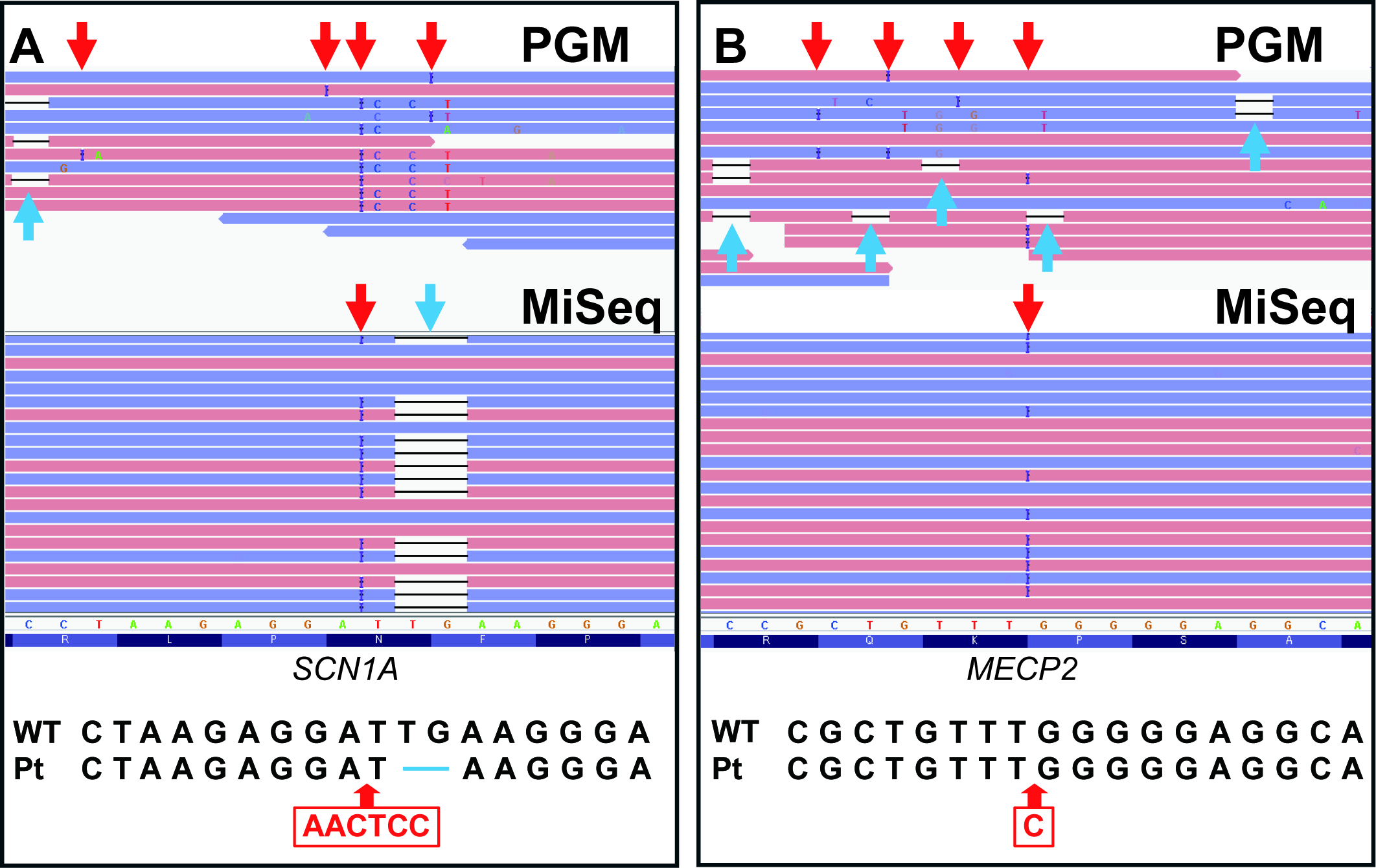

Supplement: Figure S2 — Comparison between PGM and MiSeq of mutations and sequence reads from positive control samples. (A) The c.342_344delinsAGGAGTT mutation detected in Sample 1. (B) The c.243_244insC mutation detected in Sample 7. In both panels, data was obtained from either PGM (upper) or MiSeq (lower). Both the c.342_344delinsAGGAGTT mutation and the c.243_244insC mutation were not detected in PGM with neither PGM-TMAP-Variant Caller algorithm nor PGM-Novolign-GATK algorithm. Forward and reverse read strands are shown in pink and blue, respectively. Red and blue arrows indicate insertion and deletion positions, respectively, which were confirmed by Sanger sequencing. The horizontal bar indicates the deletion call, and symbols within the read strands ( ·) indicate insertion calls detected by either PGM or MiSeq. In (A) and (B), the true inserted sequence depicted by “ ·” commonly detected by PGM and MiSeq is AACTCC and C, respectively. The DNA sequence surrounding a mutation is shown below the IGV graphics. WT, wild type; Pt, patient. (TIF) [file pone.0074167.s002.tif]
