# Supplementary material for: Performance Comparison of Bench-Top Next Generation Sequencers Using Microdroplet PCR-Based Enrichment for Targeted Sequencing in Patients with Autism Spectrum Disorder
Source: PLoS One. 2013 Sep 16;8(9):e74167. doi: 10.1371/journal.pone.0074167 (PMC3774667; doi:10.1371/journal.pone.0074167)
Supplement: Table S1 — RainDance ASDSeq™ Core Research Screening Panel. (PDF) [file pone.0074167.s003.pdf]

Table S1 RainDance ASDSeq™ Core Research Screening Panel

Gene	Chromosome
SLC2A1	1
MBD5	2
NRXN1	2
SCN1A	2
ZEB2	2
FOXP1	3
MEF2C	5
NIPBL	5
NSD1	5
BRAF	7
CNTNAP2	7
FOXP2	7
HOXA1	7
MET	7
RELN	7
CHD7	8
COH1-VPS13B	8
EHMT1	9
TSC1	9
PTEN	10
BDNF	11
DHCR7	11
FOLR1	11
AVPR1A	12
CACNA1C	12
PTPN11	12
FOXG1	14
GABRB3	15
UBE3A	15
CREBBP	16
TSC2	16
PAFAH1B1	17
RAI1	17
SLC6A4	17
TCF4	18
PNKP	19
SHANK3	22
AP1S2	X
ARX	X
ATRX	X
CASK	X
CDKL5	X
DMD	X
FGD1	X
FMR1	X
HPRT1	X
JARID1C-KDM5C	X
L1CAM	X
MECP2	X
MED12	X
MID1	X
NHS	X
NLGN3	X
NLGN4X	X
OPHN1	X
PCDH19	X
PHF6	X
PQBP1	X
PTCHD1	X
RAB39B	X
SLC9A6	X
SMC1A	X
