# Supplementary material for: Performance Comparison of Bench-Top Next Generation Sequencers Using Microdroplet PCR-Based Enrichment for Targeted Sequencing in Patients with Autism Spectrum Disorder
Source: PLoS One. 2013 Sep 16;8(9):e74167. doi: 10.1371/journal.pone.0074167 (PMC3774667; doi:10.1371/journal.pone.0074167)
Supplement: Table S2 — Summary of SNP/indel detection with PGM and MiSeq. (PDF) [file pone.0074167.s004.pdf]

Table S2 Summary of SNP/indel detection with PGM and MiSeq

	All reads				Reads with mapping quality above 40			
	Total SNPs and indels	Indels	% indels of total calls	Indels/1kb/sample	Total SNPs and indels	Indels	% indels of total calls	Indels/1kb/sample
PGM	9685	5544	57.2%	1.340	7574	5288	69.8%	1.270
MiSeq	3818	395	10.3%	0.096	3553	386	10.9%	0.093
