# Supplementary material for: Performance Comparison of Bench-Top Next Generation Sequencers Using Microdroplet PCR-Based Enrichment for Targeted Sequencing in Patients with Autism Spectrum Disorder
Source: PLoS One. 2013 Sep 16;8(9):e74167. doi: 10.1371/journal.pone.0074167 (PMC3774667; doi:10.1371/journal.pone.0074167)
Supplement: Table S3 — Summary of target resequencing and prioritization. (PDF) [file pone.0074167.s005.pdf]

Table S3 Summary of target resequencing and prioritization

	PGM	MiSeq
Total unknown variants identified from 28 patients with ASD*	98	64
Nonsynonymous SNVs	62	46
Non-segmentally duplicated SNVs	60	44
SNVs with a frequency ≤ 1% (in NHIBL ESP5400 dataset)	60	44
SNVs with a frequency ≤1% (in in-house control exomes)	57	30
True positive calls (confirmed by Sanger sequencing)	21	22
False positive calls	36	8
due to** mutation at read end	21	5
mutation near homopolymer	14	0
low depth (<10)	27	0
sequencer specific error	1	3
True positive call rate (%)	36.8%	73.3%

* After excluding low quality calls, detected variants were filtered by dbSNP135 (or lower versions of dbSNPs) which were flagged as common without clinical association.

** More thant one reason for one false positive call.
