# Supplementary material for: Performance Comparison of Bench-Top Next Generation Sequencers Using Microdroplet PCR-Based Enrichment for Targeted Sequencing in Patients with Autism Spectrum Disorder
Source: PLoS One. 2013 Sep 16;8(9):e74167. doi: 10.1371/journal.pone.0074167 (PMC3774667; doi:10.1371/journal.pone.0074167)
Supplement: Table S4 — Clinical features of patients with novel SNVs. (PDF) [file pone.0074167.s006.pdf]

Table S4 Clinical features of patients with novel SNVs

Patient	Current Age	Sex	Age at Onset	Severity*	IQ	Complications
A682	11	M	unknown	mild	93	epilepsy
A681	9	F	unknown	mild	118	epilepsy
A634	16	M	1	Severe	37	-
A447	14	M	1	Severe	32	-
A479	15	M	1	Severe	36	-
A621	12	M	1	mild	60	nocturia
A668	14	M	1	Severe	18	-
A669	6	M	1	mild	68	-
A711	8	F	1	Severe	20	sleep disorder
A464	11	M	1	Severe	75	-
A627	11	F	1	Severe	30	obesity
A651	10	M	1	Severe	30	sleep disorder
A674	6	F	1	Severe	30	-
A663	11	M	1	Severe	15	epilepsy
A653	4	M	1	Severe	40	-
A695	9	M	1	Severe	47	-
A647	9	M	1	Severe	40	-
A619	12	M	1	Severe	40	obesity

*Severity for each patient was clinically judged, referring ASD rating scales such as Social Responsiveness Scale (SRS).
