# Supplementary material for: Performance Comparison of Bench-Top Next Generation Sequencers Using Microdroplet PCR-Based Enrichment for Targeted Sequencing in Patients with Autism Spectrum Disorder
Source: PLoS One. 2013 Sep 16;8(9):e74167. doi: 10.1371/journal.pone.0074167 (PMC3774667; doi:10.1371/journal.pone.0074167)
Supplement: Table S5 — Multiple mutations detected in patients with ASD. (PDF) [file pone.0074167.s007.pdf]

Table S5 Multiple mutations detected in patients with ASD

Patient	Gene	Nucleotide : amino acid change
A663	*RAI1*	c.C1148C>T:p.P383L
	VPS13B	c.T820T>G:p.F274V
A668	*SHANK3*	c.3127C>T:p.L1043F
	DMD	c.3479A>G:p.N1160S
	DMD	c.2473A>G:p.M825V
A634	*CHD7*	c.7880G>A:p.R2627Q
	RAI1	c.4238T>C:p.M1413T
A674	*PNKP*	c.56C>T:p.A19V
	SLC6A4	c.878C>T:p.S293F
A681	*CACNA1C*	c.4706C>T:p.P1569L
	NIPBL	c.1553C>T:p.T518I
