# Supplementary material for: Performance Comparison of Bench-Top Next Generation Sequencers Using Microdroplet PCR-Based Enrichment for Targeted Sequencing in Patients with Autism Spectrum Disorder
Source: PLoS One. 2013 Sep 16;8(9):e74167. doi: 10.1371/journal.pone.0074167 (PMC3774667; doi:10.1371/journal.pone.0074167)
Supplement: Table S6 — Comparison of PGM and MiSeq analysis cost and expected yield. (PDF) [file pone.0074167.s008.pdf]

Table S6 Comparison of PGM and MiSeq analysis cost and expected yield

	Illumina MiSeq	Ion Torrent PGM
System cost	¥18,800,000	¥11,400,000
Read length (bp)	150 x 2*	100
Sequence yield per run	1.5-2G	100-200Mb **
Base call quality	>Q30	>Q20
Typical DNA requirement for sequencing	50-1000ng	100-1000ng
Run time (hr)	27	2
**In this study*** **		
Sequencing cost per sample	¥32,166	¥77,000
Sample enrichment cost per sample	¥68,000	¥68,000
Run cost per sample	¥100,166	¥145,000
Run time / sample (hr)	4.5	2

* Using MiSeq Reagent Kit (300 cycle). ** Using 316 chip. *** Six samples were multiplexed in MiSeq run.
Approximately $1=¥100
